# Fusion-Activated Ca^2+^ Entry: An “Active Zone” of Elevated Ca^2+^ during the Postfusion Stage of Lamellar Body Exocytosis in Rat Type II Pneumocytes

**DOI:** 10.1371/journal.pone.0010982

**Published:** 2010-06-08

**Authors:** Pika Miklavc, Manfred Frick, Oliver H. Wittekindt, Thomas Haller, Paul Dietl

**Affiliations:** 1 Institute of General Physiology, University of Ulm, Ulm, Germany; 2 Department of Physiology and Medical Physics, Medical University of Innsbruck, Innsbruck, Austria; University of Giessen Lung Center, Germany

## Abstract

**Background:**

Ca^2+^ is essential for vesicle fusion with the plasma membrane in virtually all types of regulated exocytoses. However, in contrast to the well-known effects of a high cytoplasmic Ca^2+^ concentration ([Ca^2+^]_c_) in the prefusion phase, the occurrence and significance of Ca^2+^ signals in the postfusion phase have not been described before.

**Methodology/Principal Findings:**

We studied isolated rat alveolar type II cells using previously developed imaging techniques. These cells release pulmonary surfactant, a complex of lipids and proteins, from secretory vesicles (lamellar bodies) in an exceptionally slow, Ca^2+^- and actin-dependent process. Measurements of fusion pore formation by darkfield scattered light intensity decrease or FM 1-43 fluorescence intensity increase were combined with analysis of [Ca^2+^]_c_ by ratiometric Fura-2 or Fluo-4 fluorescence measurements. We found that the majority of single lamellar body fusion events were followed by a transient (t_1/2_ of decay = 3.2 s) rise of localized [Ca^2+^]_c_ originating at the site of lamellar body fusion. [Ca^2+^]_c_ increase followed with a delay of ∼0.2–0.5 s (method-dependent) and in the majority of cases this signal propagated throughout the cell (at ∼10 µm/s). Removal of Ca^2+^ from, or addition of Ni^2+^ to the extracellular solution, strongly inhibited these [Ca^2+^]_c_ transients, whereas Ca^2+^ store depletion with thapsigargin had no effect. Actin-GFP fluorescence around fused LBs increased several seconds after the rise of [Ca^2+^]_c_. Both effects were reduced by the non-specific Ca^2+^ channel blocker SKF96365.

**Conclusions/Significance:**

*F*usion-*a*ctivated *C*a^2+^ *e*ntry (FACE) is a new mechanism that leads to [Ca^2+^]_c_ transients at the site of vesicle fusion. Substantial evidence from this and previous studies indicates that fusion-activated Ca^2+^ entry enhances localized surfactant release from type II cells, but it may also play a role for compensatory endocytosis and other cellular functions.

## Introduction

Ca^2+^-triggered membrane fusion is the key element of regulated exocytosis [1], [2]. Multiple Ca^2+^-dependent steps have been elucidated so far, involving molecular motors, cytoskeletal elements and SNARE proteins that finally lead to the formation of a fusion pore, an elusive, channel-like structure that represents the initial connection between vesicle lumen and extracellular space [3]–[8]. In fast secreting systems, such as the synaptic terminal, pore formation and transmitter release follow [Ca^2+^]_c_ elevations after micro to milliseconds only [4]. These systems are endowed with “active zones”, in which complex spatio-temporal [Ca^2+^]_c_ transients, determined by fixed and mobile Ca^2+^ buffers, pumps and exchangers, originate from Ca^2+^ entry through voltage-gated Ca^2+^ channels and “flood” the cytoplasm around subplasmalemmal vesicles at various stages during their rapid turnover [9], [10].

The alveolar type II cell – in contrast – can be defined as a typical “slow secreter”, according to the following features: The vesicles (lamellar bodies, LBs) are mostly very large (>1 µm), with no apparent plasmalemmal clustering but rather a scattered intracellular distribution [11]. Consequently, LBs (between zero and tens per cell) fuse in a sequential fashion for up to 20 minutes after stimulation [12]. An overall [Ca^2+^]_c_ only moderately above resting values (near 320 nmol/l) is required to induce LB fusion with the plasma membrane [13]. Voltage-gated Ca^2+^ channels are not present [14].

The postfusion phase is of particular significance for surfactant secretion: LBs contain a large and mainly water insoluble complex, where the laws of single molecule diffusion do not apply [15]. In addition, fusion pores expand slowly and are a mechanical barrier to content release [16], [17]. As a result, full content release can be further delayed for minutes to hours, and depends furthermore on an actin coat that forms around a single LB after fusion [18]. Although the mechanisms of actin coat contraction have not yet been studied in detail, Ca^2+^ is also required herein, and surfactant release cannot occur under conditions of increased intracellular Ca^2+^ buffering capacity (e.g. induced by BAPTA-AM) [18]. Although the actual physical forces (in *pN*) of the actin coat are not yet known, these features suggest the contribution of an active contraction to fully expel the vesicle contents.

Considering the delayed actions of Ca^2+^, how can agonists like ATP, which elicits a relatively short-lasting Ca^2+^ release from IP_3_-sensitive stores (via P2Y_2_ receptors and activation of PLC) [12], secrete surfactant from multiple LBs in a sequential fashion, frequently at times when the Ca^2+^ signal is already gone?

To test the hypothesis of “hidden Ca^2+^ signals”, we investigated [Ca^2+^]_c_ dynamics during all vesicle stages in more detail, taking advantage of the sequential manner of LB fusion at near basal overall [Ca^2+^]_c_ values. This enabled a mapping of individual fusion events and [Ca^2+^]_c_ on a spatial and temporal basis. Furthermore, we relied on the power of previously developed methods that allow precise and reliable determination of fusion pore formation. One of them, darkfield microscopy of scattered light intensity decrease (SLID), allows to separate the prefusion phase from hemifusion and postfusion, combined with the analysis of [Ca^2+^]_c_ during all steps [19]. The data demonstrate unambiguously that single LB fusion events are followed by a transient rise of [Ca^2+^]_c_. The possible modes of activation (mechanical or chemical) and implications of fusion-activated Ca^2+^ entry (FACE) will be discussed below.

## Materials and Methods

### Ethics statement

We isolated alveolar type II cells from male Sprague-Dawley rats (150–200 g) as described [20]. Rats were obtained from Charles River (Sulzfeld, Germany) and maintained at the central animal facility of the Ulm University according to institutional guidelines for ethical care of animals. All experiments in this study were approved by the Regierungspräsidium Tübingen, grant Nr. 833.

### Cell isolation

Rats were anesthetized (ketamin 10% and xylazil 2%), and injected with heparin (400 IU/kg). Lungs were perfused, resected, washed and incubated twice with elastase and trypsin at 37°C for 15 min. Lung tissue was immersed in DNase containing solution and sliced into ∼1 mm bits. Enzyme reaction was stopped by incubation with FCS (37°C, 2 min). The tissue was filtered 3 times through gauze and nylon meshes (mesh width: 150, 20, and 10 µm) and the final filtrate centrifuged for 8 min at 130×g. After suspending in DMEM medium, the cells were put on IgG coated plastic dishes and incubated at 37°C for 15 min. Non-adherent cells were centrifuged for 8 min at 130×g, suspended in DMEM with 10% FCS and 1% Penicillin/Streptomycin (10.000 units/ml Penicillin, 10 mg/ml Streptomycin in stock solution) and seeded on glass cover slips. Cells were cultured at 37°C, 5% CO_2_, and 95% humidity and used for experiments 1–2 days after isolation. 4 Fluo-4 experiments on the darkfield microscope were done on cells which were frozen in liquid nitrogen immediately after cell isolation and thawed 2 days before the experiment.

### Experimental conditions

Experiments were performed at room temperature in experimental bath solution (in mM: 140 NaCl, 5 KCl, 1 MgCl_2_, 2 CaCl_2_, 5 glucose, 10 Hepes; pH 7.4). Calcium free bath solution contained EGTA (2 mM) and no CaCl_2_. Lamellar body fusion was stimulated with ATP (100 µM) and phorbol 12-myristate 13-acetate (PMA; 300 nM) in darkfield/Fluo-4 and with ATP (10 µM) in FM 1-43/Fura-2 experiments, respectively. Secretagogues were added to the experimental bath solution directly, and immediately before the start of image acquisition. For the measurement of [Ca^2+^]_c_, the cells were incubated in DMEM with 5 µM Fluo-4 acetoxymethylester (AM) for 20 min or with 4 µM Fura-2 AM for 15 min. FM 1-43 was used at 2 µM in combination with Fura-2. In control experiments, cells were preincubated with CellTracker Green CMFDA (5-chloromethylfluorescein diacetate) at 800 nM for 10 minutes. To deplete Ca^2+^ stores, the cells were incubated with 150 nM thapsigargin for 20 min. In the experiments with the Ca^2+^ channel blocker SKF96365, 50 µM of this substance was either applied to the experimental bath solution directly, or the cells were additionally preincubated with it for 15 min at 37°C. OxiFluor (Oxyrase Inc., USA) was added to experimental bath (1∶200) in darkfield experiments to prevent the fluorescence-induced cell damage. Actin coating of lamellar bodies was measured in cells transfected with β-actin-GFP adenovirus construct, as described before [18]. All fluorescent dyes were purchased from Molecular Probes (Invitrogen, Germany).

### Fura-2 ratio and FM 1-43 fluorescence imaging

Measurements of FM 1-43 fluorescence in combination with Fura-2 were performed as described [20]. Using a 2-D imaging system (TILL Photonics, Germany), cells were illuminated for 20 ms at a rate of 7 Hz at each excitation wavelength (340 and 380 nm for Fura-2; 480 nm for FM 1-43). Excitation light was deflected by a 520 nm dichroic mirror. In this setting, channel crosstalk between the FM 1-43 fluorescence and the Fura-2 ratio would lead to small under-estimations of [Ca^2+^]_c_ as described in detail earlier [13]. To eliminate potential interference with Fura-2 ratio calculations, Fura-2 fluorescence was determined in ring-like ( = peri-vesicular) regions of interest (800 nm wide) surrounding the fused, FM 1-43 stained LB. Onset of the Ca^2+^ rise was specified as the time-point when the increase in the Fura-2 ratio exceeded 2-times of its SD within a period of at least 2 seconds. Ca^2+^ spreading was assessed by analyzing Fura-2 ratios (in 0.8–1 µm wide regions of interest) in 5 and 10 µm linear distances from the site of fusion for events being sufficiently separated in time to avoid overlap of spreading Ca^2+^ signals from multiple events. The onset of the Ca^2+^ rise at these locations was determined as described above for the local Ca^2+^ increase.

### Darkfield and Fluo-4 fluorescence imaging

An upright Richardson RTM 2.5 microscope (Improvision, Germany) was used for combined darkfield and fluorescence imaging. For darkfield illumination, a red LP-interference filter (>620 nm) in the condenser was used together with the RTM 3.0 blue filter cube for fluorescence imaging (490 nm dichroic, 400–500 nm excitation filter, 510 LP emission filter). Darkfield and fluorescence light was detected with a 3CCD color video camera (ExwaveHAD DXC-C33, Sony). All images were acquired with the 63× Leica water immersion objective (NA = 0.90) and Volocity software (Improvision, Germany). The darkfield and the fluorescent images were acquired simultaneously and were digitally separated based on different colors [19].

### Image analysis and data presentation

We defined a region of interest encircling the fusing LB in which darkfield light intensity change was measured. Fluo-4 fluorescence was measured within a ring-like region of interest surrounding the fusing LB. The size of selected regions of interest depended on LB size, whereas the width of the ring was set to approximately 1 µm. The lowest value of darkfield and fluorescent measurements was subtracted and the resulting change in light intensity was expressed in arbitrary units. Excel 2003 and SigmaPlot 10.0 were used for statistics and graphs. Unless otherwise stated, data are presented as mean±standard error of the mean. The alveolar type II cell photographs were corrected for brightness and contrast with Adobe Photoshop CS2. The background subtraction was made in ImageJ by subtracting the first image in an image sequence from the others with the Image Calculator feature.

## Results

**Figure 1:**
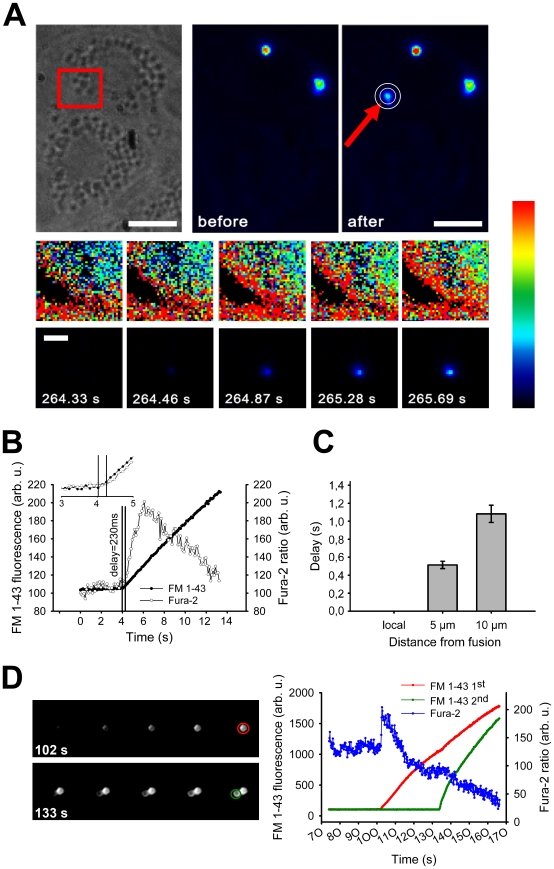
Fusion-activated Ca^2+^ entry measured by Fura-2. (A) Top: Two cells imaged by bright-field (left) and FM 1-43 fluorescence before (middle) and after (right) fusion of a single LB with the plasma membrane (arrow). Region of interest where the Fura-2 ratio was measured is indicated by the circles. Bottom: Fura-2 ratio (above) and FM 1-43 fluorescence (below) images from the area within the red square (top left) as a function of time after cell stimulation with ATP (10 µM). Scale bars: 10 µm (top) and 5 µm (bottom). (B) Time course of the Fura-2 ratio and FM 1-43 fluorescence measured around and within the area of a single LB during fusion, respectively. Inset shows detailed traces at the time of fusion. The vertical lines mark the onset of the FM 1-43 fluorescence increase and the start of the Fura-2 ratio increase. (C) Delay of onset of the Fura-2 ratio increase as a function of the distance from the site of fusion (*n* = 14). The time of the local Fura-2 ratio increase was set to 0 s and the delays 5 and 10 µm distant from the site of fusion are relative to the time point of the local increase. (D) Image sequence showing LB fusion with the plasma membrane (detected by FM 1-43 staining; upper row), followed by fusion of a second LB to the already fused one ( = compound exocytosis; bottom row). The graph on the right shows FM 1-43 fluorescence increase for both fusions (marked in red and green on the right image sequence) together with the Fura-2 ratio change around the fusing LB.

**Figure 2:**
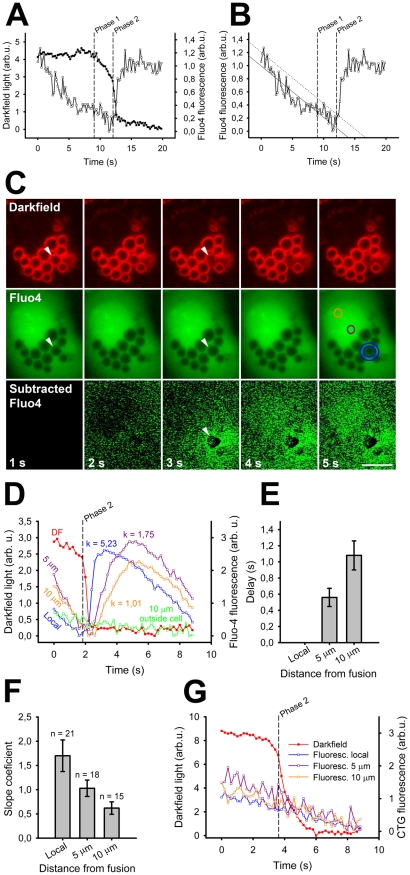
Fusion-activated Ca^2+^ entry in darkfield and Fluo-4 experiments and propagation of the local Ca^2+^ increase. (A) Decrease in darkfield light intensity at the time of fusion (filled squares) mostly occurred in 2 phases. Phase 1 (slow decrease) was shown in a previous study to reflect lipid mixing (left dashed line), phase 2 (steep decrease) was shown to reflect fusion pore opening (right dashed line). The start in Fluo-4 fluorescence increase (open squares) corresponded to the start of phase 2 in darkfield light decrease. We confirmed that the darkfield events indeed represented fusion of the LB with the plasma membrane by observing surfactant squeezing through the fusion pore in darkfield light (*n* = 12) or by subsequent staining of the fusion events with FM 1-43 (*n* = 11). (B) To determine the start of the Fluo-4 fluorescence increase the pre-fusion part of the fluorescence data was fitted by a linear regression (solid line). The first data point which exceeded the noise level of the last 2 s before the fusion (dotted line) was taken as the starting point. (C) Alveolar type II cell in darkfield and fluorescence during one LB fusion event with the plasma membrane, stimulated by ATP (100 µm) and PMA (300 nM; arrowhead). The bottom row shows the subtracted Fluo-4 fluorescence images (the first fluorescence image was subtracted from all others). Note the localized Fluo-4 increase at 3 s and the subsequent spreading over the cell. The start of the phase 2 in the darkfield recording was at 1.8 s (see graph on Figure 3D). The fusing vesicle appears darker as the others, which might happen if LBs contain a minor amount of Fluo-4, which can diffuse out of the vesicle when the fusion pore is open (see also Video S1). Scale bar = 8 µm. (D) The decrease in darkfield light intensity (red) together with Fluo-4 fluorescence measured locally (blue), 5 µm (violet) and 10 µm (orange) from the site of the fusion event shown in C. The green line indicates Fluo-4 fluorescence measured 10 µm from the vesicle outside the cell (see Figure S1). Times in D correspond to the ones in C. The dashed line indicates the start of phase 2 in the darkfield recording and k indicates the slope coefficient of Fluo-4 increase. (E) Delay of onset of the Fluo-4 fluorescence increase (*n* = 15) as a function of the distance from the site of fusion. The time of the local Fluo-4 fluorescence increase was set to 0 s and the delays 5 and 10 µm distant from the site of fusion are relative to the time point of the local increase. (F) Comparison of the slope coefficients for the Fluo-4 fluorescence increase measured at different distances from the site of fusion. (G) Example of LB fusion in an alveolar type II cell stained with CellTracker Green (CTG), a Ca^2+^ insensitive dye. CTG fluorescence around the fusing LB did not change at the time of fusion. The general decrease of fluorescence before fusion in D and G was due to photobleaching.

To investigate fusion-related Ca^2+^ dynamics in single cells, we combined two independent fusion- and Ca^2+^-indicators:

1. FM 1-43 fluorescence with the Fura-2 ratio (Figure 1)
2. SLID with Fluo-4 fluorescence (Figure 2)

In the Fura-2 experiments, LB fusion was detected with FM 1-43 added to the bath, as outlined in detail previously [16], [20]. In brief, this dye is able to diffuse from the bath through the fusion pore into the lumen of a fused LB, where the quantum yield of this dye greatly increases by its interaction with the surfactant bilayers [21]. This results in a bright localized fluorescence signal. The Fura-2 ratio was always determined in peri-vesicular regions of interest (see Materials and Methods).

**Figure 3:**
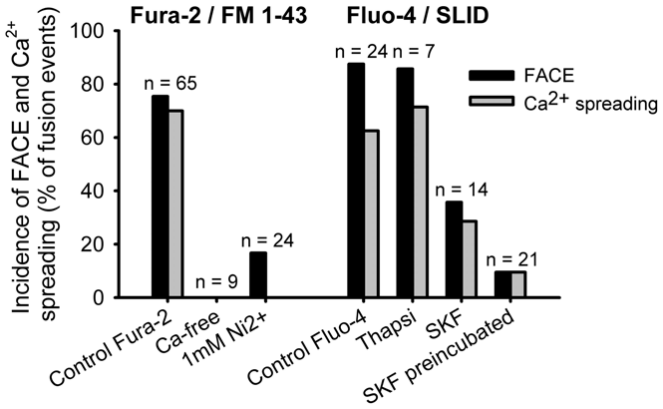
Incidence of FACE and Ca^2+^ spreading over the cell in different experimental conditions. FACE (black bars) and Ca^2+^ spreading over the cell (gray bars) were measured with Fura-2 and Fluo-4. Note that the removal of Ca^2+^ from the bath solution (Ca-free) abolished the localized Ca^2+^ increase, whereas the treatment with thapsigargin (Thapsi) did not have an effect. Treatment with nickel (1mM Ni^2+^), direct addition of the Ca^2+^ channel blocker SKF96365 (SKF), or pretreatment of the cells with it (SKF preincubated) decreased the incidence of FACE, suggesting the involvement of Ca^2+^ channels in the initiation of the localized Ca^2+^ increase. Cells were stimulated for secretion with 10 µM ATP in Fura-2 experiments and with 100 µM ATP and 300 nM PMA in Fluo-4 experiments.

The Fura-2/FM 1-43 experiments revealed the following:

1. Vesicle fusions were followed by elevations of the peri-vesicular Fura-2 ratio (Figure 1A and B), its onset being delayed with regard to the onset in increase of FM 1-43 fluorescence by 239.5±31.7 ms (*n* = 49). This indicates that a fusion pore sufficiently wide to allow FM 1-43 passage preceded the rise of [Ca^2+^]_c_.
2. The Ca^2+^ signal originated at the site of fusion but mostly (in 70% of events where such analysis was possible, *n* = 20) propagated across the cell. In this case a [Ca^2+^]_c_ rise was also detectable at a distance from the fusion site, however with a considerable delay (Figure 1C).
3. The incidence of peri-vesicular Fura-2 elevations dropped from 75.4% (*n* = 65) to 16.7% (*n* = 24) when Ca^2+^ entry was blocked in the presence of 1 mM Ni^2+^, and was completely abolished when Ca^2+^ was removed from the bath (*n* = 9; Figure 3).
4. FACE was very frequently detected in LB-plasma membrane fusions, but not in compound (LB-LB) exocytoses (see Figure 1D; *n* = 10).

The second method for detecting localized Ca^2+^ changes at the time of LB fusion was a combination of Fluo-4 fluorescence and darkfield microscopy. The latter approach, recently developed in our lab, is based on the observation that the intensity of scattered light produced by LBs decreases upon fusion with the plasma membrane [19]. For most (84.6%) vesicle fusions the decrease occurred biphasic: The first phase was shown to reflect membrane lipid merger, whereas the second corresponded to FM 1-43 fluorescence increase and opening of the fusion pore [19]. In the current study we measured the darkfield light intensity together with Fluo-4 fluorescence.

The peri-vesicular increase of the Ca^2+^ concentration was measured in a circular 1 µm wide region of interest (Figure 2C). The start of the Fluo-4 fluorescence onset was determined by fitting the last 3–12 seconds of the pre-fusion trace, and the first data point above the noise level in the last 2 seconds of the Fluo-4 recording was taken as the start of fluorescence increase (Figure 2A and B). Localized increase in [Ca^2+^]_c_ was detected in 87.5% of fusion events (*n* = 24; 21 experiments, 13 cell preparations) and initiated after the start of the second, steep phase of the scattered light intensity decrease (SLID2). Previous work showed that the start of the SLID2 corresponded to the fusion pore opening [19], therefore the combination of darkfield microscopy and Fluo-4 fluorescence lead to the same conclusion as the combination of FM 1-43 fluorescence and Fura-2 ratio. The mean delay between the onset of the SLID2 and initiation of localized Fluo-4 fluorescence increase was 0.59 s±0.40 (mean±SD; *n* = 21) which was longer than the delay between the FM 1-43 fluorescence and the Fura-2 ratio increase. Possible reasons for this discrepancy could be:

- different methods to analyze the instance of fusion pore opening. Previous work revealed that the onset of FM 1-43 fluorescence may be delayed by up to 0.5 s with regard to SLID2 or cell membrane capacitance steps, respectively [22]. Although occasions of strongly limited FM 1-43 diffusion through the pore are not frequent, they tend to underestimate the delay between pore opening and onset of a Ca^2+^ signal.
- different affinities of Fluo-4 (K_d_ = 345 nM) and Fura-2 (K_d_ = 224 nM) for Ca^2+^ ions
- relatively poor signal to noise ratio in most Fluo-4 experiments and ensuing uncertainties regarding the precise determination of a starting point of FACE (Figure 2B).

The localized post-fusion increase in [Ca^2+^]_c_ was transient (Figure 1B and 2D), the median half-time of the decrease in Fluo-4 fluorescence experiments was 3.2 s (interquartile range 2.1–6.5 s; *n* = 19). The maximum observed half-time of decrease was 26.6 s. The major reason for the fast Fluo-4 fluorescence decrease in most experiments is probably photobleaching, which was present already before the fusion event (Figures 2A and D).

In the majority of fusion events (62.5%), Ca^2+^ spread over the entire cell (Figure 2C and Video S1). The onset of the [Ca^2+^]_c_ increase was measured locally, and 5 and 10 µm away from the fused LB. The mean delay between onset of SLID2 and that of the [Ca^2+^]_c_ increase was 1.13 s and 1.65 s at 5 and 10 µm, respectively (*n* = 15, Figure 2E). The calculated mean velocity of [Ca^2+^]_c_ spreading was therefore 9.43 µm/s, which is consistent with the reported slow diffusion of Ca^2+^ ions in the cytoplasm [23] and confirms results obtained in Fura-2 measurements (10.82 µm/s; calculated from data in Figure 1C).

An additional indication that a Ca^2+^ wave propagated throughout the cytoplasm starting from one location (the fused LB), was the measurement of the slope coefficients for the Fluo-4 fluorescence increase. If the [Ca^2+^]_c_ increase is actually starting from a point source in the cell, we would expect these slope coefficients to be steepest close to the Ca^2+^ source and less steep with increasing distance. A global [Ca^2+^]_c_ increase (as caused by a homogeneous release from stores) should result in similar slope coefficients throughout the cell. The slope coefficients, calculated from linear regressions, for the local [Ca^2+^]_c_ increase were significantly higher (1.70±1.49; mean±SD; *n* = 21) than those of the [Ca^2+^]_c_ increase 5 µm (1.03±0.72; mean±SD; *n* = 18), and 10 µm away from the fused LB (0.62±0.50; mean±SD; *n* = 15; paired t-test, p<0.01; Figure 2F).

To ensure that the localized increase in Fluo-4 fluorescence was not caused by dye redistribution (although a very unlikely possibility), we stained the cells with CellTracker Green (CTG), a cytoplasmic dye, which is not Ca^2+^ sensitive and has a molecular weight that is even smaller than that of Fluo-4 (465 compared to 1097 Da). In measurements combined with darkfield we did not observe an increase in CTG fluorescence in the vicinity of an LB at the time of fusion (measured in concentric areas as in Fluo-4 experiments), nor a change in fluorescence 5 and 10 µm away (*n* = 8; 5 experiments, 4 cell preparations; Figure 2G).

The first question from these experiments is the subcellular source or even molecular identity of this localized [Ca^2+^]_c_ increase. Fura-2 fluorescence experiments in the absence of bath Ca^2+^ showed the absolute requirement of extracellular Ca^2+^ for the initiation of the localized [Ca^2+^]_c_ increase and for the Ca^2+^ spreading (Figure 3). To additionally investigate the possible involvement of intracellular Ca^2+^ stores, cells were incubated with thapsigargin, which inhibits the Ca^2+^-ATPase on the endoplasmic reticulum, thereby depleting internal Ca^2+^ stores [24]. To test the efficiency of thapsigargin treatment we added it (150 nmol/l) directly to the experimental bath solution under the microscope. ATP stimulation after 20 min caused no increase in [Ca^2+^]_c_ indicating depleted stores. However, in cells treated with thapsigargin, 85.7% of vesicle fusions (n = 7; 6 experiments, 3 cell preparations) were still accompanied by a localized [Ca^2+^]_c_ increase (in control conditions 87.5%), which excludes the major role of the endoplasmic reticulum in initiation and propagation of the Ca^2+^ increase (Figure 3, note that cells had to be stimulated by ATP and PMA: PMA causes LB fusion without a rise of [Ca^2+^]_c_ [25]). On the other hand, the thapsigargin experiments cannot exclude the contribution of Ca^2+^ ions from IP_3_-insensitive stores, possibly LBs themselves: they have high Ca^2+^ concentrations, especially those located near the apical membrane [26]. Ca^2+^ transport into LBs is most likely caused by Ca^2+^/H^+^ and Ca^2+^/K^+^ exchangers, whereas the Ca^2+^-ATPase doesn't seem to play a major role [27]. Therefore, Ca^2+^ release by LBs in thapsigargin-treated cells cannot be entirely ruled out. However, since the removal of bath Ca^2+^ abolished the postfusion [Ca^2+^]_c_ increase measured by Fura-2, it is evident that intracellular stores (IP_3_ sensitive and –insensitive) do not contribute to this phenomenon.

**Figure 4:**
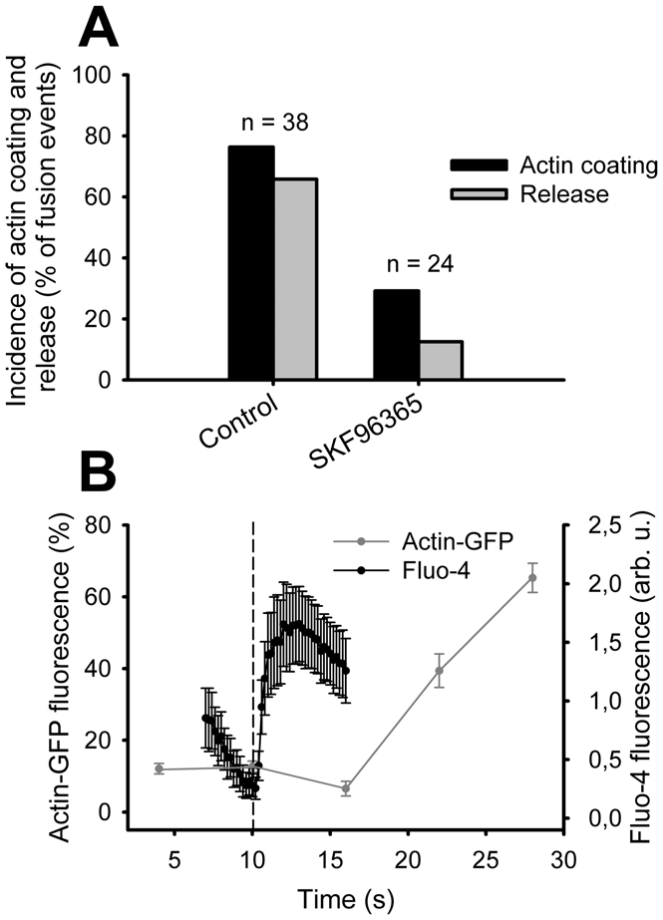
Correlation between post-fusion actin coating and localized Ca^2+^ entry. (A) Incidence of fusion events followed by actin coating and surfactant release in control conditions and after addition of SKF96365 to the bath. (B) The temporal relationship between localized Ca^2+^ increase and actin coating of the fused vesicles. Fluo-4 fluorescence data (*n* = 11) were acquired at 5/s and the actin-GFP fluorescence of fusing vesicles (*n* = 29) at 10/min. The start of the darkfield light decrease (designating fusion pore opening) was set to the time point 10 s (dashed line). Cells were stimulated for secretion with 100 µM ATP and 300 nM PMA added directly to the experimental bath.

Previous work showed that the cytoplasmic Ca^2+^ chelator BAPTA-AM inhibited the actin coat formation around fused vesicles, as well as surfactant release from LBs [18]. This observation strongly suggests that localized [Ca^2+^]_c_ increase may assist in actin coat formation and contraction in the course of surfactant secretion. To test the possible connection between the post-fusion [Ca^2+^]_c_ increase and the actin coating we tried to block the post-fusion [Ca^2+^]_c_ increase with the unspecific Ca^2+^ channel blocker SKF96365. By direct application of SKF96365 to the cells, the incidence of the localized post-fusion Fluo-4 fluorescence increase dropped from 87.7% to 35.7% (*n* = 14; 9 experiments, 3 cell preparations, Figure 3). Likewise, in cells transfected with actin-GFP the incidence of post-fusion actin coating decreased from 76.3% (*n* = 38; 13 experiments, 10 cell preparations) to 29.2% (*n* = 24; 8 experiments, 5 cell preparations, Figure 4A). Pre-incubation of cells with SKF96365 for 15 min at 37°C further decreased the incidence of post-fusion [Ca^2+^]_c_ increase to 9.5% (*n* = 21; 11 experiments, 5 cell preparations; Figure 3), suggesting that Ca^2+^ enters the cytoplasm through channels. This conclusion is further strengthened by the observation that 1mM Ni^2+^, a non-specific cation channel blocker, also decreased the incidence of post-fusion [Ca^2+^]_c_ increase to 16.7%. The start of the post-fusion Ca^2+^ influx preceded the start of actin coating for several seconds (Figure 4B), which excludes the possibility that the opening of the Ca^2+^ channels was caused by rearrangement of actin cytoskeleton.

## Discussion

### Methodological aspects

The major new finding of this study is that exocytotic fusion can trigger Ca^2+^ entry. At first glance this is a paradox situation and contradicts common textbook knowledge that fusion is triggered *by* Ca^2+^, including the surfactant secreting alveolar type II cell. This prompts the devil's advocate to ask the following questions:

- Could our estimations of the instance of fusion pore formation and rise of [Ca^2+^]_c_ be so imprecise that “hidden [Ca^2+^]_c_ elevations” prior to fusion are constantly overseen?
- Could the [Ca^2+^]_c_ transients, that we observe, be just random Ca^2+^ release events, similar to Ca^2+^ sparks in muscle cells, with no causal relation to LB fusion?

If Ca^2+^ sparks existed in alveolar type II cells, and if our methods were indeed imprecise, the notion that random Ca^2+^ sparks trigger LB fusion events would – in principle – be possible. However, apart from the fact that our estimates of fusion pore formation rather tend to underestimate than overestimate the delay between fusion and [Ca^2+^]_c_ rise (see Results), the following arguments exclude such a scenario:

1. Spontaneous [Ca^2+^]_c_ elevations in the absence of LB fusion events were essentially never observed. Hence, there is no evidence for the existence of spontaneous, Ca^2+^ release events in alveolar type II cells.
2. Alveolar type II cells have no readily releasable vesicle pool [13], [20]. When cells are stimulated by flash photolysis of caged Ca^2+^, creating a uniform Ca^2+^ elevation, the earliest LB fusion events occur several seconds after the [Ca^2+^]_c_ rise [13]. Hence, even if “Ca^2+^ sparks” existed in type II cells, they would not be able to trigger LB fusion right away.
3. The peak of peri-vesicular [Ca^2+^]_c_ increase can be delayed with respect to fusion pore opening for several seconds (Figure 1B, 2A and 2D). Even if a minor, undetected increase in [Ca^2+^]_c_ would exist already before fusion pore opening, the plain fact that the major [Ca^2+^]_c_ elevation occurs in the postfusion phase raises questions about the origin, activation and possible role of this phenomenon.

It is also important to consider in this context that an artifactual misinterpretation caused by possible dye accumulation in LBs, which would measure extracellular Ca^2+^ following fusion pore formation, can be excluded for the following reasons:

1. The signal spread within the borders of a cell exclusively, and not into the outside of the cell, as would be the case if Ca^2+^-sensitive dye was secreted along with surfactant material (see also Figure S1).
2. LBs contain a high (mM) Ca^2+^ concentration prior to fusion [26]. Hence, in case of dye accumulation, they should appear bright, not dark, excluding a possibility that the LBs contain large quantities of the dye.
3. Video S1 clearly demonstrates that the fluorescence intensity increase after fusion occured around, not within, LBs.

### Origin of FACE

**Figure 5:**
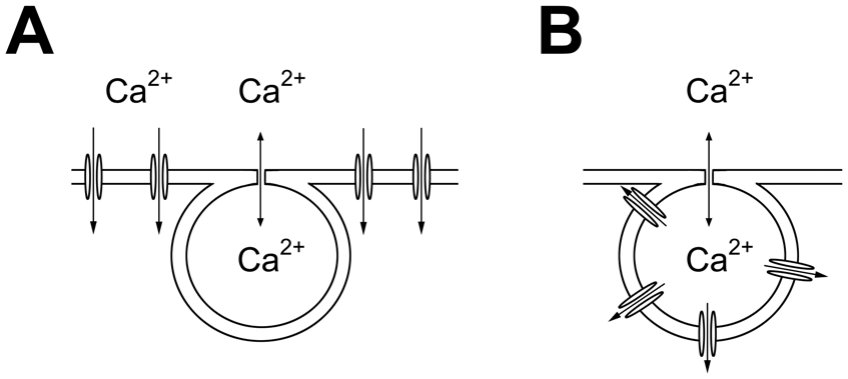
Possible pathways of FACE. (A) Ca^2+^ entry into the cytoplasm through channels located on the plasma membrane. (B) Ca^2+^ entry into the cytoplasm through channels located on the LB membrane. Here Ca^2+^ entry occurs in series with the fusion pore, with the consequence that the amount of Ca^2+^ entering the cytosol could be a function of fusion pore permeability, LB membrane permeability and amount of Ca^2+^ ions stored inside the LB. In both cases, A and B, channel activation may occur mechanically (membrane stress) or chemically (membrane merger; see also Discussion). The relative contribution of each model to FACE is yet unknown. In addition, intracellular amplification of the FACE-induced Ca^2+^ signal by Ca^2+^-induced Ca^2+^ release may take place.

Less clear than the mere fact that fusion triggers [Ca^2+^]_c_ elevations, is the question of its origin: does Ca^2+^ come from the extracellular space exclusively, or also from the fusing LB? However, the distinction between LB and extracellular space is - in this context - somewhat semantic, because during the postfusion phase, the lumen of the LB is by definition part of the extracellular space (see below and Figure 5).

A similar problem refers to the notion of “Ca^2+^ entry” vs. “Ca^2+^ release”. Conventionally, “Ca^2+^ entry” refers to Ca^2+^ entering the cytoplasm from the extracellular space, whereas Ca^2+^ release denotes Ca^2+^ coming from intracellular stores. Naturally, IP_3_-insensitive Ca^2+^ stores may exist that also contribute to the [Ca^2+^]_c_ rise, including the LB itself. However, considering all evidence (abolishment by removal of bath Ca^2+^, ineffectiveness of thapsigargin), it is reasonable to assume that the crucial origin of Ca^2+^ is the extracellular space, for which “fusion-activated Ca^2+^ entry” (FACE) is the appropriate term. Ca^2+^-induced Ca^2+^ release may be an amplification mechanism that applies particularly for those cells, in which the signal spreads to sites remote from the location of fusion.

Recently, Chintagari et al. [28] showed that inhibition by bafilomycin A1 of the V-ATPase on the LB membrane causes Ca^2+^ release from LBs. If Ca^2+^ stored in LBs was the major source of FACE, it should be greatly blocked by this drug, which depletes this store. However, we found that pretreatment with bafilomycin A1 (20 nM for 1h at 37°C) could not inhibit FACE (3 of 4 fusion events were followed by FACE, data not shown). This further strengthens the notion that the major part of Ca^2+^ comes from the extracellular space (possibly through the fused LB, see Figure 5), a pathway for which the term “Ca^2+^ entry” is most appropriate.

### Pathway(s) and mode of activation of FACE

This is largely a matter of speculation. FACE appears to be independent of the mode of cell stimulation: It occurred after submaximal stimulation with ATP, after maximal stimulation with ATP plus phorbol ester (see above) and also after stimulation of protein kinase A (with 20 µM terbutaline and 5 µM forskolin, in 3 of 4 fusion events, data not shown). Evidence suggesting that an ion channel is involved arises from the time-dependent inhibition by SKF96365 and from inhibition by Ni^2+^ ions. SKF96365 is a broad inhibitor of various TRP channels [29]–[32]. Preincubation-time dependent inhibition in fact suggests that the majority of FACE does not occur through the plasma membrane, but through the limiting membrane of LB. If Ca^2+^ entered through the plasma membrane, the effect of SKF96365 should not depend so strictly on its incubation time. The slow action rather suggests a long diffusion path to reach a channel inside the LB. On the other hand, Ni^2+^ is not membrane permeable and should only block those channels that are directly exposed to the extracellular space. Both inhibitors had a strong effect on the incidence of FACE, however possible differences in their targets as well as the observation that none of them could completely inhibit FACE, do not allow for a clear conclusion about the nature and localization of putative Ca^2+^ channels.

Also speculative are possible modes of activation of FACE. Two mechanisms are most likely:

- mechanical (“membrane stress”)
- chemical (“membrane merger”)

#### Mechanical activation

The strongest argument in favor of a mechanical mode of activation is LB swelling. Many secretory vesicles swell after fusion through osmotic uptake of water by the vesicle matrix [33], and postfusion LB swelling is considerable, with volume increases up to >20% [19]. However, postfusion vesicle swelling is not necessarily associated with stretch (and stress) of its limiting membrane, because membrane tension can cause a replenishing lipid flow from the plasma membrane to the vesicle membrane, increasing the membrane area [34]. The membrane stress hypothesis is therefore conceivable under either of the following conditions:

1. The fusion pore is a significant barrier for free lipid flow from the plasma into the LB membrane, such that swelling-induced LB membrane stress occurs. Recent observations of lipid-anchored fluorophore diffusion measurements suggest that this may be the case [19].
2. LB swelling leads to ion channel activation by channel-cytoskeleton interactions.

Since both conditions are possible, mechanical activation of FACE is a reasonable working hypothesis.

#### Chemical activation

Recent studies indicate that lipid merger between the plasma and vesicle membranes cause translocation of lipid mediators and activation of signaling cascades that finally induce actin assembly [35]–[37]. Any of such locally formed signaling molecules could – in theory – cause the activation of a Ca^2+^ channel.

### Possible functions of FACE

Since the [Ca^2+^]_c_ elevation caused by FACE in most cases spread out through the entire cell (see Results), the whole spectrum of Ca^2+^-dependent functions could be envisaged. Owing to its localized origin, however, and a large body of additional evidence, the following 3 functions are most likely for alveolar type II cells:

- Actin coat formation and surfactant release
- Endocytosis
- Activation of adjacent LBs, possibly for inducing compound exocytosis

#### Actin coat formation and surfactant release

Awareness is growing that postfusion regulation of content release may be relevant to cell physiology in various types of secretion (recently reviewed in Ref. [38]). Beside the known effects of Ca^2+^ and protein kinase C on fusion pore expansion [16], [39], recent evidence supports a role of molecular motors, such as F-actin and myosin 2 phosphorylation, for fusion pore and vesicle dynamics [38]. We have previously shown that single LB fusion events with the plasma membrane trigger the formation of a locally restricted actin-GFP coat around the fused LB [18]. This coat is likely necessary for LB “contraction” and surfactant release into the extracellular space (the term “contraction” denotes an apparent squeezing-out of surfactant through the fusion pore, although contractile forces of this coat have not yet been directly measured). Since actin coat formation occurs clearly after FACE (Figure 4B), a dependence of actin coat formation (and surfactant release) on FACE (and not vice versa) is strongly suggested. We therefore aimed at further testing this hypothesis applying a non-specific, broadly acting Ca^2+^ channel inhibitor (SKF96365). Inhibition of both, FACE and actin coat formation, by this drug strengthens the notion that a major physiological role of FACE could be the facilitation of LB contraction and surfactant release. This function may gain particular importance if the fusion pore does not fully expand and remains a physical barrier for release, as demonstrated earlier [16], [17]. Experiments elevating [Ca^2+^]_c_ with the Ca^2+^ ionophore ionomycin showed that fusion pore expansion is enhanced by an increased Ca^2+^ concentration [16]. Therefore it is conceivable that FACE could also promote the fusion pore enlargement and content release. The mechanisms of actin coat formation are complex and have been discussed in detail by others [40]. However, the role of Ca^2+^ and the identity of molecular effectors involved in this process remain an open question. In addition, FACE may not only contribute to actin assembly, but also to active force generation and content release.

#### Endocytosis

The compensatory membrane retrieval after vesicle fusion is thought to depend on cytoplasmic Ca^2+^ (e.g. [41]). In previous work on postfusion actin coating, we demonstrated that the whole LB together with its contents can be retrieved (‘kiss and run’ events), which was not observed under conditions of Ca^2+^ chelation with BAPTA-AM. Hence, kiss-and-run events might be influenced by FACE as well. In see urchin eggs Ca^2+^ influx necessary for endocytosis was mediated by P-type Ca^2+^ channels [42]. However, this mechanism is unlikely in type II pneumocytes, because they lack voltage-gated Ca^2+^ currents, in resting state as well as during all stages of exocytosis [14].

#### Activation of neighboring LB fusion

Since the threshold [Ca^2+^]_c_ for LB fusion with the plasma membrane is very low [13], FACE should be able to contribute to LB fusions. In alveolar type II cells, subsequent LB fusions at sites very close to each other are frequently observed, and cell degranulations often appear to proceed like a “chain reaction” rather than a simultaneous event [25], consistent with other cell types [43]. It is not clear, if homotypic LB-LB fusion is activated by Ca^2+^ to the same extent as heterotypic LB-plasma membrane fusion. Nevertheless, the localized origin of FACE in conjunction with the strong Ca^2+^-dependence of LB fusion with the plasma membrane strongly suggests that FACE stimulates subsequent LB fusions at a closely located site. Hence, FACE could be a mechanism which contributes to increased, localized secretion, possibly compound exocytosis.

### Summary

In this study, we have identified a new Ca^2+^ entry mechanism (FACE). Its molecular pathway(s) and mode(s) of activation remain to be elucidated. Owing to common principles of exocytosis (membrane merger, vesicle swelling, etc.), FACE may also play an important role in other secretory cell types, in which time and space-resolved resolution of exocytotic fusion and Ca^2+^ dynamics are more difficult to detect than in the pulmonary alveolar type II cells.

## Supporting Information

Figure S1 Fluo-4 fluorescence increase after vesicle fusion was only detected on the area of the exocytosing cell. Left: Regions of Fluo-4 measurements. Orange circle: within the cell, green circle: outside the cell (see main text and Figure 2C). The fusing vesicle is marked by an arrow. Right: Fluorescence change within both areas at the time of fusion. There was a considerable increase in intracellular Fluo-4 fluorescence but no increase at the same distance from the fusing vesicle outside the cell. Therefore, the fluorescence signal was caused by intracellular spreading of Ca2+ and not by redistribution of extracellular dye, excluding a possible misinterpretation of the data.
(0.90 MB TIF)

Video S1 Subtracted Fluo-4 fluorescence video for the fusion event shown in Figure 2C and D. The video is 9 s long and encompasses the time window shown in Figure 2D. The first, subtracted, black image in the image sequence is not shown. Note the localized nature of the fluorescence increase at the time of fusion and the subsequent spreading over the cell.
(6.60 MB AVI)
