# Supplementary figures and images for: Fusion-Activated Ca2+ Entry: An “Active Zone” of Elevated Ca2+ during the Postfusion Stage of Lamellar Body Exocytosis in Rat Type II Pneumocytes
Source: PLoS One. 2010 Jun 8;5(6):e10982. doi: 10.1371/journal.pone.0010982 (PMC2882333; doi:10.1371/journal.pone.0010982)

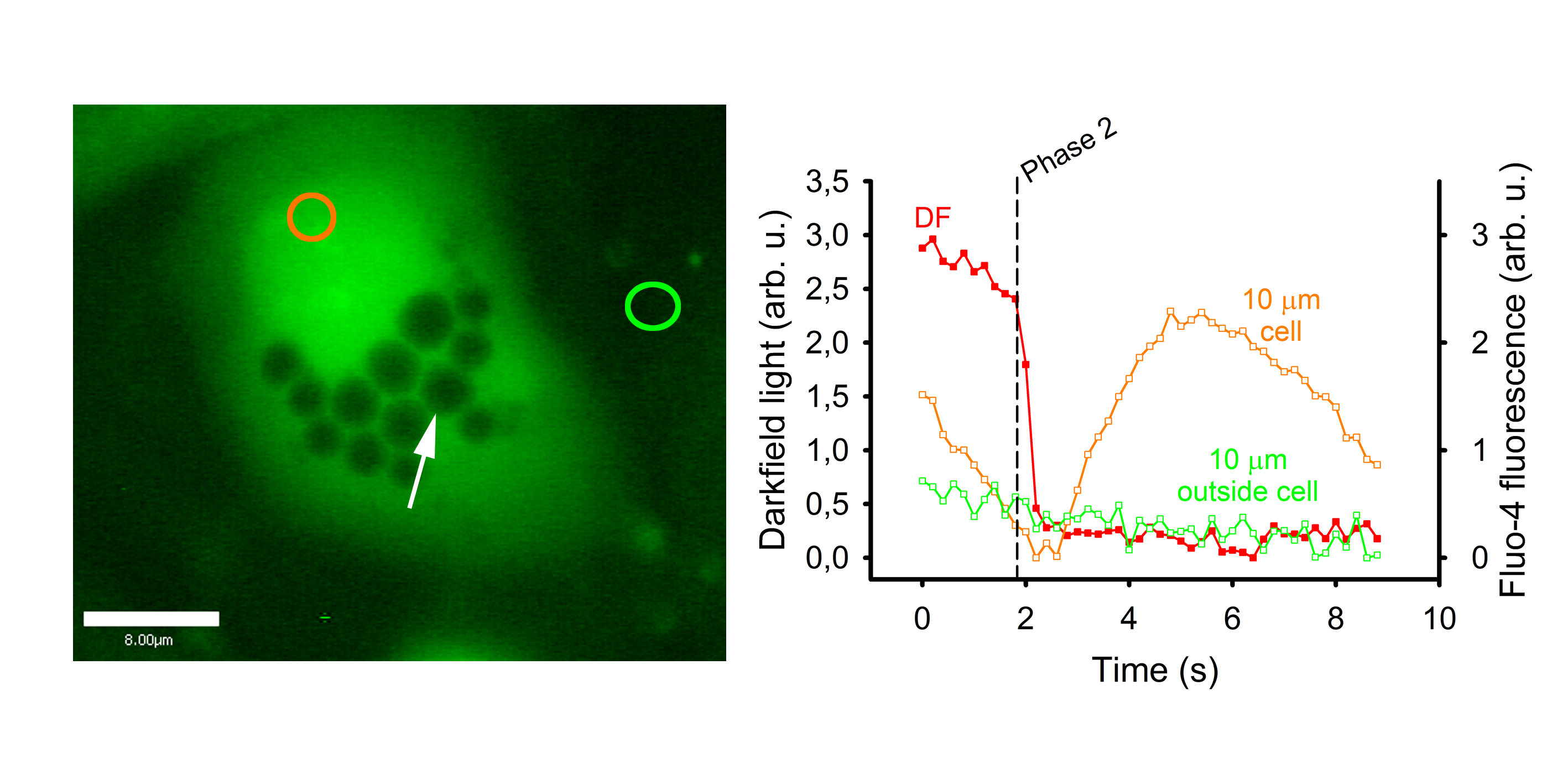

Supplement: Figure S1 — Fluo-4 fluorescence increase after vesicle fusion was only detected on the area of the exocytosing cell. Left: Regions of Fluo-4 measurements. Orange circle: within the cell, green circle: outside the cell (see main text and Figure 2C). The fusing vesicle is marked by an arrow. Right: Fluorescence change within both areas at the time of fusion. There was a considerable increase in intracellular Fluo-4 fluorescence but no increase at the same distance from the fusing vesicle outside the cell. Therefore, the fluorescence signal was caused by intracellular spreading of Ca2+ and not by redistribution of extracellular dye, excluding a possible misinterpretation of the data. (0.90 MB TIF) [file pone.0010982.s001.tif]
